# The link between emotion comprehension and cognitive perspective taking in theory of mind (ToM): a study of preschool children

**DOI:** 10.3389/fpsyg.2023.1150959

**Published:** 2023-05-10

**Authors:** Krystian Macheta, Arkadiusz Gut, Francisco Pons

**Affiliations:** ^1^Institute of Psychology, Pedagogical University of Kraków, Kraków, Poland; ^2^Department of Cognitive Science, Nicolaus Copernicus University in Toruń, Toruń, Poland; ^3^Department of Psychology, University of Oslo, Oslo, Norway

**Keywords:** emotion understanding, theory of mind, opacity, language, emotions

## Abstract

The study examined the relation between perspective taking embedded in theory of mind (ToM) and emotion comprehension (EC) in young children. Our study involved children from Poland aged 3–6 (*N* = 99; 54% boys) from public and private kindergartens residing mainly in urban areas, whose parents could mostly be classified as middle class. The children were examined with the Test of Emotion Comprehension (TEC) and three tasks targeting three aspects of ToM: a first-order false belief task, an appearance-reality test, and a mental states opacity task. The results showed similarities in performances between these different measures. However, only the opacity task predicted the emotion comprehension test results (*η*^2^ = 0.13). The results indicate that the key element of ToM that explains individual differences in children’s emotion comprehension is the full-blown understanding of perspective taking, namely that having access to an object under one description does not ensure access to that object under all descriptions. In the research, we took also into account the linguistic side of such specific competences as ToM and EC, which allowed us to see the role of language in scaffolding the development of children’s ability to handle such socially fundamental tasks as understanding emotions and epistemic states.

## Introduction

Emotion comprehension (EC), as the declarative dimension of emotional competence, might be defined as the capacity to understand the nature, causes and consequences of emotions, its main function being to allow the child to identify, explain, and predict emotions, to control the expression of emotions, and to regulate the experience of emotions (Cutting and Dunn, 1999; Conte et al., 2019; Pons and Harris, 2019; Sarmento-Henrique et al., 2020).

The ability to talk about emotions emerges in children around their second year of life. When it first appears, children already talk about both their own as well as others’ emotions, though children’s own emotions receive much more attention. The lexicon used by children to talk about emotions at that age is fairly limited, but it does include words for both negative and positive emotions (Harris et al., 2016). Interestingly, expressions that describe behavior associated with emotions, such as “to laugh” or “cry,” emerge earlier than words for the corresponding emotions—“happiness” and “sadness” (Storm and Storm, 1987; Terwgot and Stegge, 2001). A more advanced form of emotion comprehension emerges in three-year-olds. They are capable of understanding that someone else can feel an emotion that is different from their own, even though the situation of that other person is similar to the child’s own. Later, as the child develops, understanding of emotions becomes increasingly complex; for instance, child starts to appreciate that more than just one emotion can be felt at the same time (Pons et al., 2004).

Theory of mind (ToM), on the other hand, is the ability to identify mental states of others as well as one’s own (Wimmer and Perner, 1983; Wellman et al., 2001). Most generally, ToM is responsible for understanding that different people may hold different beliefs, including false beliefs or beliefs that are contrary to reality (Gopnik, 2011; Flavell et al., 1990; Mitchell and Lacohée, 1991; Perner et al., 1987; Perner and Lang, 2000). The capacity emerges in humans at a relatively early stage and seems not to require any previous training. Children that use ToM are claimed to explain other people’s behavior by attributing to them various mental, unobservable states.

We have to remember, however, that there is a significant group of studies that have demonstrated a cross-cultural variance in ToM performance, and now there is substantial data available from a number of different cultures (Gut and Mirski, 2016; Dixson et al., 2018). Children shift to above-chance performance in ToM at around 6 years old in Japan (Naito, 2014). In Pacific cultures, such as Samoa (Mayer and Träuble, 2012) and Vanuatu (Dixson et al., 2018), the age is roughly 8 years. Similarly, Nawaz et al. (2014) demonstrated a lag for Pakistani children compared to Western samples where 4-year-olds still performed at chance. Further, while Chinese children from mainland China perform similarly to their Western counterparts, children from Hong Kong demonstrate a 2-year lag, starting to pass false belief task (FBT) around 6 (see a large meta-analysis in Liu et al., 2008). We point out this cross-cultural variance in ToM performance to emphasize that in our study, we included children from a Western culture, who typically pass FBT at around 4 years old, and equally importantly, these children are usually exposed to social experiences that involve consideration of minds and their states (Jakubowska et al., 2018; Gut et al., 2020).

Research on emotion understanding in children also indicates cross-cultural variation. For example, a cross-cultural study of Chinese preschool children (Tang et al., 2018) showed that although an understanding of emotions develops in Chinese children according to the same pattern found in European American preschoolers, at a more nuanced level, Chinese preschoolers performed better at understanding hidden emotions compared to European children. Other studies indicate that in addition to cultural factors, socioeconomic status (SES) also has a strong impact on the development of emotion understanding (Kårstad et al., 2016).

Considerable research efforts have been devoted to investigating the relation between ToM and EC (e.g., Denham, 1986; Hughes and Dunn, 1998; Cutting and Dunn, 1999; Eggum et al., 2011; Weimer et al., 2012). According to many researchers, an association between ToM and EC is strongly expected (Cutting and Dunn, 1999; Lane et al., 2010; Eggum et al., 2011; Sarmento-Henrique et al., 2020). The arguments for that are that both ToM and EC: (a) make use of the same cognitive resources (e.g., general intelligence); (b) are implicated in language in important ways; (c) involve the same specific competences (e.g., decoupling mental states from reality, perspective taking), which—importantly—emerge in children around the same age; (d) the social environment is likely putting the need to differentiate mental states and emotions at around the same time in development (Hughes and Dunn, 1998; Weimer et al., 2012; Grazzani et al., 2018; Conte et al., 2019).

Despite multiple studies, a consensus has not been reached on what exactly the relation between ToM and EC is (Sarmento-Henrique et al., 2020). Some studies showed that children start to label mental states related to emotions at the age of 2–3 years (I am, for example, happy or angry), before being able to label cognitive mental states (I see, I know, I want) at around the age of 3–4 years (Wellman et al., 2001). Other studies showed that children first come to understand the distinction between the appearance and reality of physical objects at the age of 4–5 years and then of emotions at the age of 6–7 years (Gross and Harris, 1988; Harwood and Farrar, 2006; Bjørk et al., 2022; Lombardi et al., 2022). Numerous studies have shown that children understand ignorance and false beliefs at the age of 5–6 years before being able to understand the impact of ignorance and false beliefs on emotions at the age of 6–7 years (de Rosnay et al., 2004; Bender et al., 2011). Researchers claim that the data gathered so far support both an important link between ToM and EC, as well as their potential independence (e.g., Dunn, 1995; Conte et al., 2019). Even though most researchers emphasize that children’s understanding of others’ minds is closely related to their development of emotional competence (Ensor et al., 2011; Conte et al., 2019), it is still unclear what exactly makes the two competences related to each other. One possibility is a lack of methodological precision, such as drawing conclusions about the construct of ToM without paying much attention to the particular ways it is measured (Ornaghi et al., 2016; Sarmento-Henrique et al., 2020). Another issue may be significant cultural variations in the understanding of ToM or emotions in different cultures, as indicated by the studies of Liu et al. (2008) and Mayer and Träuble (2012).

Accepting the claim that a relation between ToM and EC is likely (Harwood and Farrar, 2006; Bender et al., 2011; Weimer et al., 2012; Kuhnert et al., 2017), we wish to find out which of the elements of the two competences are responsible for the relation. Strictly, we aim to establish which of the elements of the ToM construct contributes to explaining the variance in EC; that is, which of the elements that constitute the child’s ability to attribute epistemic states is central for EC as well.

To discover which of the elements of the ToM construct contributes to explaining the variance in EC, we first need to begin from the fact that there are a number of tasks that have been designed to study the ToM construct, its functioning and developmental sequences involved. The most prominent of these tasks is the false-belief test (FBT), which measures the child’s ability to understand that people can have false beliefs and that they are guided by those beliefs in action, believing them to be true. Based on numerous studies conducted so far, the FBT is considered a highly robust measure of ToM and is often referred to as the litmus test for understanding other minds (Wimmer and Perner, 1983; Wellman et al., 2001). Customarily, it was assumed that once the child passes FBT, he/she possesses an almost full-blown concept of mental representation and a fully-fledged representational theory of mind, which includes understanding opacity (see, for example, Gopnik, 1993; de Villiers and Pyers, 2002). Importantly, there is significant correlation between FBT and other measures designed to study other aspects of ToM: the appearance-reality test (understanding that something can appear to be something different than it actually is; Flavell, 1993), the pseudonym test (Doherty and Perner, 1998), and the say-something-different test (Perner et al., 2002, 2003). Considering these correlations, a number of researchers began to assume that passing FBT attests to the child’s understanding of the basic intuitions inherent not only to the appearance-reality distinction but also to the concept of opacity (Gopnik et al., 1999). By that rationale, children who use the mechanism of theory of mind while correctly attributing a false belief *ipso facto* understand that substitutions of extensionally identical terms in propositions preceded by an intentional term (think, know, believe) are not guaranteed to preserve the truth value of the type of a sentence: “I know-think that p.” In other words, for some researchers a representational understanding of mind implies a perspectival understanding of mind (Perner et al., 2002, 2003). Hence the idea that once the child passes FBT, he/she possesses an almost full-blown concept of mental representation and a fully-fledged representational theory of mind.

Despite these claims, at least two issues need to be addressed in the context of the relation between ToM and EC. First, even though there may be strong correlation between ToM tasks, it should be kept in mind that they still measure different aspects of the ability. One of the tests, which was designed to tap into a slightly different aspect of ToM, is the appearance-reality task (ART). The creator of the task, Flavell (1993), states that in the case of appearance-reality, young children need to differentiate real vs. apparent object identity (for example, a sponge that looks like a rock), which requires a conceptual understanding that there can be a difference between how something seems or appears to be and how something really is (Flavell, 1993). Unlike FBT, ART requires that children understand that the human mind may represent objects in different ways, especially that something can appear to be something different than it actually is. It seems that children understand faked emotions around age 4 (Sidera et al., 2012).

Second, passing FBT does not terminate the development of socio-cognitive competences that fall under the ambit of ToM. In order to understand how ToM skills unfold, it is critical to examine children’s performance on the intensionality or opacity task (OT). Its aim is to measure yet another aspect of ToM: the understanding that the fundamental characteristic of mental states is that they represent under particular descriptions and not others, and the epistemic consequences that follow from this inherent perspectivity of our mind (Russell, 1987; Gopnik et al., 1999; Apperly and Robinson, 2001, 2003; Rakoczy et al., 2015). The opacity task (OT) typically involves the scenario in which the protagonist of a story or situation is acquainted with one of a few valid descriptions of a given object or person, whereas the examined child also knows an alternative description or descriptions of the same object or person. For example, in one of the first studies in the field (Apperly and Robinson, 2001), children were presented with several items: a bouncy ball, a rubber dice, and some other objects, which were contained in similar tin boxes; and a puppet called Heinz, who was the protagonist in the covering story. Each of the objects was referred to by two possible definite descriptions: “ball/present,” “dice/rubber” etc. (Apperly and Robinson, 2001). Answering the substitution-sensitive question, like “Does Heinz know that there’s an Y (e.g., a present) in the box?,” [that is such question in which one should not replace X (ball) with Y (present), even though it is true that Y = X (e.g. ball is a present for someone)], most of the children of 4 or 5 years accepted substitution, making evident that they did not understand the opacity of intensional contexts.

Studies using OT have shown that children of 4–5 years still have difficulty understanding what opacity and intensionality of mental states consist of (Kamawar and Olson, 1999, 2011; Apperly and Robinson, 2001, 2003; Hulme et al., 2003; Proft et al., 2019; Schroeder et al., 2021). It can be stated, then, that regardless of the form of the task, the research revealed that children who reliably passed the FBT still may exhibit difficulties handling a task in which they are confronted with different versions of the opacity test (Apperly and Robinson, 2001, 2003; Gut et al., 2020).

In light of the above results, it seems that ToM continues to develop even after the FBT has been passed. Further, it remains within the realm of possibility that after passing FBT, the child’s theory of mind unfolds in a piecemeal fashion. If so, then there would be neither a radical change (one conceptual system for another), nor a shift from a two-stage representation (semantics) to a three-stage representation (semantics). Rather, the gap in performance would involve a conceptual enrichment that consist in expanding the content of particular concepts (e.g., “belief”), enabling the child to add new, more sophisticated principles of mental states attribution to already existing abilities. We can term it a transition from coarse-grained content to fine-grained content. Such a hypothesis would lead to the interpretation that false-belief reasoning, appearance-reality reasoning and opacity reasoning all involve multiple perspectives. However, in the case of FBT, the perspective taking involves truth value (true-correct vs. false-incorrect). ART, on the other hand, involves understanding of the difference between how things appear and how they really are. Finally, in the case of OT, the perspective taking focuses on the mode of presentation—it is possible in this test that two different conceptualizations or descriptions can both be true.

In order to pass OT, children have to realize some new aspects of how beliefs work: namely, in OT, the child needs to understand that having access to an object under one description does not ensure access to that object under all the other descriptions. It has therefore turned out that the opacity problem properly adapted as a task testing children’s understanding of the opacity of mental states “differs crucially from that of a partially informed protagonist in appearance-reality tasks or standard false belief tasks” (Apperly and Robinson, 2001, p. 375). It should also be noted that even though ART and FBT measure different aspects of ToM, they do share the general characteristic that the child needs to understand that partial informational access can lead to misidentification of an object (e.g., of a sponge as a rock, or of the pencils contained in a sweet tube as sweets), whereas in the opacity task (OT) the protagonist holds true, but limited knowledge about the object, possessing one of many *true* conceptualizations.

Considering the three tasks (FBT, ART, OT) together, we can say that ToM needs to include knowledge that beliefs can be true or false (i.e., fitting the world or not). It also includes understanding of the difference between appearance and reality, and that appearances can be misleading, even though they can be the basis for forming a belief. Finally, ToM also involves the knowledge that access to an object under one conceptualization does not entail access to it under another conceptualization, even when both of those conceptualizations are true.

To fully understand the research design of the present study, it is important to stress that the emotion understanding in question here is not only the ability to ascribe emotions to people based on their facial expressions, or the understanding that certain events evoke their related emotions. Rather, the present study addresses that two people can feel two different emotions about the same situation, and that the same person can feel two different emotions at the same time about some situation (i.e., mixed emotions). All of these seem to play a role in a more sophisticated ability to attribute emotions, which requires perspective taking skills (cf. Harwood and Farrar, 2006). For that reason, in the present study we aim to discover which of the studied kinds of perspective taking is most significant in understanding and attributing emotions.

In consequence, even though the three aspects of mental-state understanding are part of the overarching construct of ToM, they can be dealt with separately, and measured separately, which is done with the use of the FBT, ART, and OT. So, in our study, we wish to see which of the aspects of ToM links with EC. For that reason, apart from employing the test of emotion comprehension (TEC), we also use the three tests from the ToM family that test mental-state understanding from different perspectives. This will allow us to demonstrate the strength of the relationship between each of the tests and TEC, which we believe can help us make a more precise claim as to what about ToM links the most with understanding emotions (EC).

Prior research shows that the observed improvement in emotion understanding and perspective taking (i.e., in taking into account the other’s perspective—ToM) is often linked with the increase in language ability (Tager-Flusberg, 2000; Pons et al., 2003; Astington and Baird, 2005; Beck et al., 2012; Austin et al., 2014; De Stasio et al., 2014; Hinzen et al., 2014; Hinzen, 2017; Conte et al., 2019; de Villiers, 2020; Shahaeian et al., 2023). Bearing this in mind, as well as the key role of language in the development of both ToM and EC, we decided to pay attention to the linguistic knowledge of participating children. Yet, we will seek to determine if a subtler way of understanding mental states by children (as found in research on ToM using three tasks), which is linked with emotion understanding, is related to language ability in the same way as to emotion understanding. Focusing on the linguistic side of such specific competences as ToM and EC will allow us to define the role of language in scaffolding the development of children’s ability to handle such socially fundamental tasks as understanding emotions and epistemic states.

## Methods

### Participants

Ninety-nine Polish children aged 3–6 participated in the study (*M* = 4.59, *SD* = 1.1; 54% were boys). The study included 21 three-year-olds (*M* = 41.76, *SD* = 2.51, 52.4% boys), 25 four-year-olds (*M* = 53.59, *SD* = 2.95, 52.0% boys), 27 five-year-olds (*M* = 64.93, *SD* = 3.41, 55.6% girls) and 26 six-year-olds (*M* = 76.15, *SD* = 3.01, 65.4% boys). The children were recruited from kindergartens around Poland and were from working or middle-class socio-economic backgrounds. Around 30% came from rural areas. They were assessed by their parents/tutors as children within the developmental norm.

### Measures

#### Test of emotion comprehension

Test of Emotion Comprehension (TEC) is a tool created in 2000 (Pons and Harris) to measure children’s comprehension of emotions. TEC is related to basic skills of recognizing emotions, understanding the impact of situations and beliefs on emotions. It also focuses on one’s understanding of the relations between emotions and memories, beliefs, and hiding of an experienced emotion. Moreover, is related to such processes as emotion regulation, mixed emotions, and morality. Solving each of the tasks gives the child one point (minimum score = 0, maximum score = 9). The nine components of TEC are hierarchically related to one another. TEC has proven psychometric properties both in Western and non-Western cultures (Cavioni et al., 2020). We used the Polish version of TEC adapted to Polish conditions and language. Previous studies in Poland have shown (Stępień-Nycz, 2014) that the Polish version of TEC, just like its original counterpart (Pons and Harris, 2000), is a tool that allows to capture child emotion understanding and measure nine components of emotion understanding: (1) emotion recognition, (2) external cause, (3) desire, (4) belief, (5) reminder, (6) regulation, (7) hidden, (8) mixed, and (9) morally based emotions. At the same time, it enables the distinction of three phases of emotion understanding: the external phase, the mental phase, and the reflective phase. The Polish translation used in the presented research has satisfactory reliability (*α* = 0.73).

#### False belief task

At the beginning of the task, two experimenters introduced themselves to the child. Then, the experimenters and the child sat at the table in the room. A popular box of eight Kinder chocolate bars was used. However, instead of chocolates, there were colored pencils in the box. The first experimenter checked whether the child was familiar with the chocolates, asking who usually bought the chocolates in the child’s house and whether the child liked them. A control question was also asked: “*Do you know what’s usually in the box?*.” After the child answered, the first experimenter and the child participant remained in the room whereas the second experimenter left the room, saying that she would be back in a while. After the third person had left the room, the first experimenter showed the content of the box to the participant, and it turned out that there were pencils in the box, not chocolates. Then, the experimenter asked the child the test question: “*Does X who has left the room think that there are pencils in the box?*” (where X stands for the name of the second experimenter). If the child answered no, he/she scored 1, otherwise he/she scored 0. No other scores were possible.

#### Appearance-reality task

At the beginning of the task, two experimenters introduced themselves to the child. Then, the experimenters and the child sat at the table in the room. Next, an object that looked either like a lollipop or a rock was produced from a box by one of the experimenters. The child was then asked: looking at the object that I’m holding in my hand, can you tell me what it is? Children who correctly identified the object as a lollipop or a rock moved on to the next stage of the study. After the child answered, the first experimenter and the child participant remained in the room whereas the second experimenter left the room. After the third person had left the room, the first experimenter revealed what the object really was; the child could touch it and see that it is really an eraser (in the lollipop version) or a sponge (in the rock version). After the child said what the object was, the experimenter asked the test question: What will the person who just left (using their first name), who could not touch the object, think the object is when they come back? If the child answered either lollipop or rock depending on the version of the task, he/she scored 1, otherwise he/she scored 0. No other scores were possible.

#### Opacity task

Two experimenters introduced themselves to the child, and then they sat at the table in the room. A plastic toy car that was also a ballpoint pen was used; the pen was hidden and would slide out after pressing a button. The first experimenter took out the car from a black box. The child could touch the car and play with it for a minute. The second experimenter participated in the play. Next, the first experimenter put the car back into the box, and the second experimenter left the room. After that, the experimenter took out the car again, telling the child that she was going to show him/her something extra. The experimenter pressed the hidden button on the car, which made the pen slide out. The child could draw something with it on a sheet of paper, after which the experimenter made the pen slide back in and put the car-pen back into the box. Then, the first experimenter asked the child two questions: “*Does X, who has left the room, know that there is a pen in the box?*,” and *“Does X, who has left the room know that there is a car in the box?*” (where X stands for the name of the second experimenter). The first question was the main test of opacity understanding. If the participant understood opacity, he/she would answer “*no*” and scored 1, otherwise he/she scored 0. No other scores were possible. The second question was the main control question. If the child had correctly assigned knowledge to the protagonist, he/she would answer “*yes.*” These two questions were asked in random order.

Additionally, considering that the relation between EC and ToM can be mediated by linguistic competence (e.g., Pons et al., 2003), general intelligence (e.g., Albanese et al., 2010), which also improve significantly around that age, we have included these variables in our analysis.

*Raven’s progressive matrices* test was first described in 1938 (Raven, 1941) and its colored version, which we used in our study, was developed in 1947. The test measures non-verbal intelligence with 36 tasks broken down into three 12-task series, with no time limits (Raven, 2000). In each of the tests, the child is asked to indicate the missing fragment of a bigger picture from a set of possibilities. Each correct answer is scored with one point. In our study, we used a paper version of the test.

*Verbal ability—RVT* receptive vocabulary test (*Obrazkowy Test Słownikowy, OTSR*; Haman and Fronczyk, 2012) is a test with a growing level of difficulty that contains 88 questions, each scoring one point. The test measures the child’s understanding of nouns, adjectives, and verbs. Each question consists of four pictures, only one of which depicts the word that the experimenter is asking for. The child’s task is to point to the right picture.

## Procedure

Two separate, quiet rooms in pre-schools were dedicated to the study. Each child did the tasks individually: the ToM tasks in one room, and the Test of Emotion Comprehension (Pons and Harris, 2000), Receptive vocabulary test OTSR (Haman and Fronczyk, 2012), and colored Raven progressive matrices in the Polish adaptation (Jaworska and Szustrowa, 1993) in the other room.

Based on the literature and previous research presented in the introduction, our strategy for analyzing data and presenting results was as follows. Firstly, we checked for statistically significant differences between the ToM task group, specifically FBT, AR, and OT. Next, we examined whether children who performed well on the ToM tasks showed differences in language skills, intelligence, and emotion comprehension compared to children who did poorly on the ToM tasks. Based on the literature, we expected to find differences between FBT and OT. Additionally, we expected the OT task, due to its specificity, to be strongly associated with emotion comprehension, and children’s language abilities to be crucial to this task. To deepen our analyses, children were divided into two groups (1) children who passed OT and (2) children who did not pass OT, and differences in means were verified between these groups and their performance in emotion comprehension, on the language skill task. Then, to determine whether language abilities predicted the correct execution of the OT task, logistic regression analysis with 20,000 bootstraps and a 95% confidence interval was performed. Next, we examined whether correct performance on the OT task significantly predicted the results of the emotion comprehension test, while checking whether the FBT and AR tasks were significant predictors of emotion comprehension. These expectations were verified using backward elimination regression analysis. Keeping in mind the expected key role of language, we attempted to examine whether language ability mediated the relationship between age (in months) and the ability to understand emotions, through a mediation analysis using the PROCESS v.4.1 macro (Model 4; Hayes, 2013).

## Results

Considering all the kids, we obtained statistically significant differences between performance on different the ToM tasks *χ*^2^ = 12.42, *p* = 0.006. Further analysis using *χ*^2^ with adjustment of *p*-value by Bonferonni method for column proportions demonstrated significant differences between the opacity task (OT) (50.5% children answered correctly) and FBT (*χ*^2^ = 11.01, *p* < 0.001) and ART (67.7%, *p* = 0.016). No statistically significant differences were found between FBT and ART and between ART and OT. Moreover, the significant age-related difference, tested with *χ*^2^, appeared in OT (*χ*^2^ = 12.42, *p* = 0.006). Differences between age groups in FBT and AR tasks were not significant. Detailed descriptive statistics for ToM tasks are included in the Table 1.^1^ There were no gender differences in ToM skills (non-significant *χ*^2^ for FTB, AR and OT)—both by age group and when analyzing all respondents combined.

**Table 1:** The sum and percentage of correct answers in theory of mind tasks.

Variable	Age (*N* and % of correct answers)				
	3	4	5	6	All
FBT	10 (47.6%)	16 (64.0%)	22 (81.5%)	20 (76.9%)	68 (68.7%)
AR	13 (61.9%)	14 (56.0%)	21 (77.8%)	19 (73.1%)	67 (67.7%)
OT	6 (28.6%)	10 (40.0%)	14 (51.9%)	20 (76.9%)	50 (50.5%)

FBT, first order false belief task; AR, appearance-reality task; OT, opacity task.

We tried to establish whether the children who did well on the ToM tasks show differences in their performance in language skill, intelligence, and emotion comprehension from the children who did poorly (see Table 2).

**Table 2:** Descriptive statistics of the tests.

Variable	Age (*M* and *SD*)				
	3	4	5	6	All
TEC	3.24 (0.83)	3.92 (1.78)	4.85 (1.73)	5.81 (1.55)	4.53 (1.80)
OTSR	32.86 (15.82)	52.64 (14.29)	59.00 (17.36)	69.23 (14.55)	54.54 (19.99)
RAVEN	9.95 (3.46)	14.24 (3.47)	17.19 (3.21)	19.88 (4.93)	15.62 (5.21)

TEC, test of emotion comprehension; OTSR, receptive vocabulary test; RAVEN, Raven’s progressive matrices test.

It was revealed that there was no significant difference between the children who did well on FBT or ART and those who did poorly in their performance on the emotion comprehension test, language skill test and intelligence test. However, there was a significant difference found in emotion comprehension (*t*(97) = 2.50, *p* = 0.014, *d* = 0.49) between those children who passed the OT task (*M* = 4.96, *SD* = 1.62) and those who did not (*M* = 4.08, *SD* = 1.88). Children who passed the OT task (*M* = 61.10, *SD* = 16.35) and those who did not (*M* = 47.84, *SD* = 21.26) also differed significantly in their performance on the language skill task (*t*(90.10) = 3.47, *p* = 0.001, *d* = 0.66).

Importantly, logistic regression analysis with 20,000 bootstraps and a 95% confidence interval was used to estimate the effects to reveal the role of language. The analysis showed that language ability significantly predicts OT (i.e., the ability to discern the intensions/aspects under which one object is known): (*b* = 0.02, CI [0.01–0.04], *p* < 0.001). Finally, there was a significant difference in the intelligence test results (*t*(97) = 2.05, *p* = 0.044, *d* = 0.40) between children who passed the OT task (*M* = 16.66, *SD* = 4.70) and those who did not (*M* = 14.55, *SD* = 5.53).

In order to test whether the emotion comprehension results can be predicted on the basis of children’s ToM results (FBT, ART, OT), linear regression using backward elimination was conducted.

Model 3 turned out to significantly predict the emotion comprehension test results *F*(1,97) = 14.83; *p* < 0.001; *η*^2^ = 0.13. The value of parameter *b*_1_ was 1.31; *p* < 0.001. This means that the only significant predictor of the emotion comprehension test results was the opacity task (OT). The beta parameter indicated that the score on the task OT is a significant positive predictor of emotion understanding and explains 12.4% of TEC variance. FBT and ART did not make it into the final model (see Table 3).

**Table 3:** Linear regression using backward elimination analysis for TEC as the dependent variable.

Model		Unstandardized coefficients		Standardized coefficients	*T*	Significance	95% CI	
		*B*	Std. Error	Beta			Lower	Upper
1	(Constant)	3.62	0.34		10.77	<0.001	2.95	4.28
	FBT	0.26	0.37	0.07	0.70	0.484	−0.48	1.00
	ART	0.16	0.37	0.05	0.45	0.657	−0.57	0.90
	OT	1.21	0.35	0.34	3.43	0.001	0.51	1.92
2	(Constant)	3.68	0.30		12.20	<0.001	3.081	4.28
	FBT	0.31	0.36	0.08	0.86	0.390	−0.40	1.02
	OT	1.23	0.35	0.34	3.52	0.001	0.54	1.93
3	(Constant)	3.83	0.25		15.42	<0.001	3.33	4.32
	OT	1.31	0.34	0.36	3.85	<0.001	0.63	1.98

CI, confidence interval; FBT, first order false belief task; AR, appearance-reality task; OT, opacity task.

**Figure 1:**
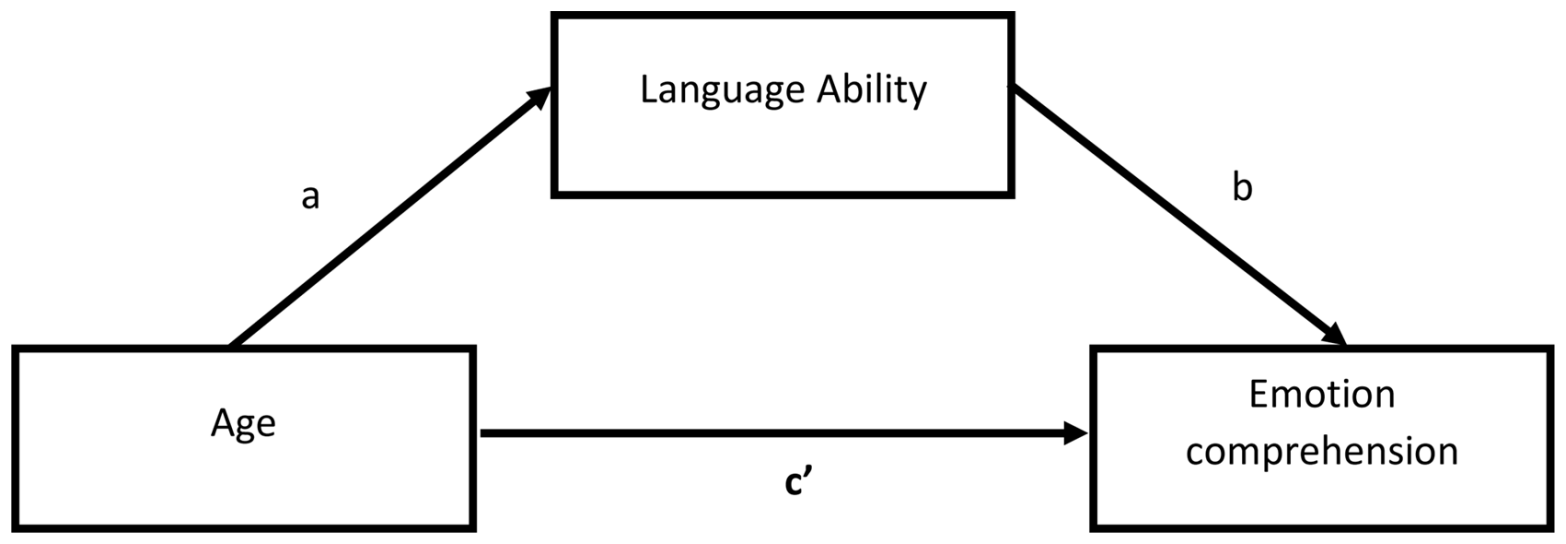
General mediation model. Meaning of symbols a,b,c’ as in description below the Model Table.

**Figure 2:**
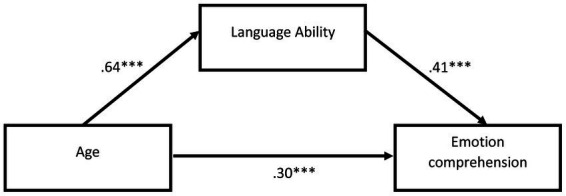
General mediation model. Path coefficients were standardized; **p < 0.01, ***p < 0.001. The correlation between age and emotion comprehension without mediation is 0.55, *p* < 0.001.

In terms of the role of language, which significantly predicted children’s understanding of the opacity of mental states, we examined whether or not language ability is a mediator between age (in months) and the ability to understand emotions, through a mediation analysis using the PROCESS v.4.1 macro (Model 4; Hayes, 2013). A regression model with 20,000 bootstraps and a 95% confidence interval was used to estimate the effects. Age significantly predicted the level of linguistic ability (*b* = 1.03, *p* < 0.001, 95% CI [0.79–1.27]), which was positively related to the ability to understand emotions (*b* = 0.03, *p* < 0.001, 95% CI [0.21–0.47]). There was also a statistically significant mediating effect between age expressed in months and the ability to understand emotions by language ability (*b* = 0.03, 95% CI [0.02–0.05]). When the mediator was included, the direct effect between age and language ability was reduced from *b* = 0.07, *p* < 0.001, 95% CI [0.05–0.09] to *b* = 0.03, *p* = 0.003, 95% CI [0.01–0.05]. This model explains 48% of the variance (see Table 4 and Figures 1, 2).

**Table 4:** Values of the mediation model.

Model	*R*^2^	*c*′	*A*	*B*	*ab*	95% CI	
						Lower	Upper
Age-LA-EC	0.48***	0.03**	1.03***	0.03***	0.03	0.02	0.05

Age, age expressed in months; LA, language ability; EC, emotion comprehension; *c*′ = direct effect of predictor on dependent variable including mediator; *a* = effect of predictor on mediator; *b* = effect of mediator on dependent variable; *ab* = indirect effect of predictor on dependent variable using mediator; *R*^2^ = variance explained by model; CI = confidence intervals for interaction *ab*. **p < 0.01, ***p < 0.001.

## Discussion

The main goal of the study was to see which of the aspects of ToM is the one that links with emotion comprehension. The link between opacity and emotion understanding that our study has identified suggests that perspective taking understood in term of mode of presentation is crucial for both understanding beliefs and emotion. As we have stressed in the introduction, success on OT requires the competence of distinguishing the intensions under which the object is known, not just knowing if the person registered the object or not. The child needs to consider the perspective or intention under which a given object in belief is presented, not only epistemic value of the belief. The central understanding that is required by the task is therefore that we can conceptualize objects from different but equally legitimate perspectives and that access to an object under one conceptualization does not entail access under another conceptualization.

The above considerations allow us to answer the question which of the different ‘mindreading’ skills is central to emotion comprehension, and by the same token to confirm the hypothesis proposed by Harwood and Farrar (2006): “theory of mind performance should be most strongly linked to the social and emotional awareness used for completing tasks such as affective perspective taking” (p. 402). The strong link between children’s understanding of opacity and their understanding of emotions that our study has confirmed agrees with the view that “affective perspective taking reflects the ability to recognize the emotional state of someone else, particularly when it differs from the person’s own emotional state” (Cutting and Dunn, 1999; Harwood and Farrar, 2006, p. 402). Perspective taking understood in terms of the mode of presentation is therefore the common denominator that is key for both belief understanding and emotion comprehension—not so much in their basic versions as in their more advanced forms.

Further, it needs to be noted that emotions are not a property of particular situations inasmuch as they are a way of reacting to them. They are what we may call a “mode of reaction.” This is analogous to the mode of presentation that the OT revolves around. Importantly, when we are talking about a mode of presentation, we do not talk about different properties of the object or its aspects, but rather about the content of the representation. In intensional contexts, when we ascribe a thought or belief to someone, the description (identification) of the object must be constrained by the knowledge held by the person. It therefore seems that understanding both emotion and opacity require an understanding of the mode of relation to the situation or some object; that is, a point of view, an aspectual shape. The mode of presentation is not directly based on the epistemic evaluation of something as true or false, or correct or incorrect.

Such understanding of perspective taking allows children to comprehend that it is entirely legitimate to feel two different emotions (e.g., happy and scares) in the same situation. What allows it seems to be the ability to understand different modes of presentation of the situation. For instance, a child can be happy when her parents promise to take her bungee jumping, which she has always dreamt of, but the prospect of the jump might also evoke fear in her. This is a typical mixed emotion situation, which forms part of TEC and which is one of the most important aspects of understanding emotions generally.

Examples of mixed emotions can also be found in the stories in Peng et al. (1992), where “the protagonist’s mother would not buy a T-shirt which the protagonist really wanted but she did buy a new pair of shoes which the protagonist liked” which were used in the original study to teach children that one can be sad and happy at the same time. The central point here is that if the child is unable to recognize that not only one of those emotions is felt at the same time but both are, she misses a more nuanced outlook on the situation, such where the emotions mix. Children in Peng et al. (1992) considered only one mode of presentation (not buying the T-shirt), but ignored the other (the bought shoes). Such ignorance means that the child has partial knowledge, but not false one (like in FBT). Neither does she identify the object mistakenly as is the case in ART where a sponge is taken for a rock.

The ignorance discussed above is an example of partial knowledge tested in OT. The child who says that the protagonist is sad ignores the fact that the protagonist got the shoes from her mother as well, which should evoke positive emotions. However, the child still uses a true belief—the desired T-shirt indeed was not bought. It is only when both of these facts—the child not getting the T-shirt, but getting the shoes—are considered jointly can we talk about the child attributing the protagonist a set of mixed emotions.

One task from TEC is in a way analogous to OT. There, the child is required to understand that the amount of knowledge that the protagonist has might evoke two emotions. The main and important difference is that in TEC one emotion is felt by the observer of the scene and follows from her partial knowledge; while the other emotion is felt by the protagonist and follows from her partial knowledge.

The child sees that the wolf is watching the bunny, which can cause the child to feel fear that the wolf can attack the bunny. It is a legitimate emotion in this situation. At the same time, however, the bunny has her back turned to the wolf and does not see him. She is oblivious to the presence of the wolf and continues eating her favorite carrot, feeling happy. The happiness of the bunny is also a legitimate emotion considering what we know about the situation. Both the child’s fear and the bunny’s happiness are legitimate in the situation. The reason for that is that they link with the respective knowledge of the child and the bunny. The task therefore requires the child to differentiate between two different perspectives, linking them to two states of knowledge, which allows children to fully grasp the emotional state experienced by another person. Neither of the emotions and their corresponding epistemic perspectives is more legitimate than the other. This is analogous to the task in OT, where the content of the propositional attitude has to be specified in terms of a point of view, partial information access, or mode of presentation, but not based only on the epistemic evaluation of something as true or false. Only by deploying the category of a mode of presentation—linked with the relevant partial knowledge—can the child distinguish two different, but equally legitimate ways of knowing the same situation. Then the child can associate them with the relevant two conflicting emotions and infer that had the bunny known that he was being observed by the fox, his happiness of eating the carrot would have also been accompanied by fear.

In order to show competencies from domain general, we examined children’s intelligence and language skills. Language skills were found to play a significant role in understanding mental states (what is required in the OT task where children have to think in a much subtler way, considering the intension under which a given object is presented) and in a multidimensional understanding of emotions (what is tested in TEC tasks). Following de Villiers (2020) and Hinzen (2017), we showed—on the one hand—that language skills significantly predict understanding OT and—on the other hand—that they significantly mediate the relationship between age and the ability to understand emotions. In both cases, language plays an important role in developing these two competences. Future research should explore the role of various aspects of language skills not only as prerequisites for the development of social skills, but also as interconnectors of such social skills.

Due to the cross-cultural variance in ToM performance for children aged 3–8 years old, described in the introduction, we must keep in mind that the findings from our study apply to a group of Western children. These are children who typically pass tasks such as FBT at the age of four, and children whose parents, as indicated by research conducted in Poland, have a strong tendency to introduce mental utterances, both affective and cognitive, in their conversations and storytelling with children (Jakubowska et al., 2018). Thus, the observed frequent use of mental vocabulary (affective and cognitive) by parents in the ethnic group we studied, as reported by Jakubowska et al. (2018), may be regarded as a form of cultural and environmental scaffolding of advanced epistemic and emotion understanding, which we see in the children in our study.

There are some limitations to the presented study. One is that we used only three tests measuring ToM, which is only a small subset of the tasks used in the literature. These include, for instance, the second-order FBT and the Silent Films task designed by Rory Devine and Hughes (2013), or the Faux Pas Recognition Test, each of which revolves around the child listening to short stories and being asked to predict and explain the characters’ behavior or thinking. Using these tasks to study children aged 7 and older could throw light on how later stages of ToM development relate to emotion comprehension. Based on the literature indicating that SES (i.e., parental education and self-reported family economy) can be a crucial factor enhancing children’s development in the area of emotional understanding and ToM, future studies should collect very detailed data regarding parents’ economic status, in addition to general information about the group (Raval and Walker, 2019; Bjørk et al., 2022).

## Data availability statement

The raw data supporting the conclusions of this article will be made available by the authors, upon request to the corresponding author.

## Ethics statement

The studies involving human participants were reviewed and approved by the Komisja Etyki Badań Naukowych, Katolicki Uniwersytet Lubelski Jana Pawła II. Written informed consent to participate in this study was provided by the participants’ legal guardian/next of kin.

## Author contributions

KM and AG research planning. KM and AG conducting research, database organization. KM statistical analysis. KM, AG, and FP preparation of the article plan and writing the manuscript. All authors contributed to the article and approved the submitted version.

## Funding

This work was supported by the Emerging Field: Perception, Cognition, and Language in the frame of Excellence Initiative—Research University Programme at Nicolaus Copernicus University to AG and by the National Science Centre in Poland grant (project number 2020/37/N/HS6/00779) to KM.

## Conflict of interest

The authors declare that the research was conducted in the absence of any commercial or financial relationships that could be construed as a potential conflict of interest.

## Publisher’s note

All claims expressed in this article are solely those of the authors and do not necessarily represent those of their affiliated organizations, or those of the publisher, the editors and the reviewers. Any product that may be evaluated in this article, or claim that may be made by its manufacturer, is not guaranteed or endorsed by the publisher.

## Footnotes

^1^Table 1 is intended to illustrate the sum and percentage of correct answers in the theory of mind tasks by age group. However, we have more precise data—the age of the respondents in months, which allows (if assumptions are met) the use of parametric tests. We decided to take advantage of this opportunity because parametric tests require less data to make a stronger conclusion than nonparametric tests (Kaur and Kumar, 2015), and parametric tests usually have higher statistical power than nonparametric tests (Grech and Calleja, 2018).
